# Robotic-assisted versus conventional laparoscopic-assisted total gastrectomy with D2 lymphadenectomy for advanced gastric cancer: short-term outcomes at a mono-institution

**DOI:** 10.1186/s12893-019-0549-x

**Published:** 2019-07-09

**Authors:** Shan-ping Ye, Jun Shi, Dong-ning Liu, Qun-guang Jiang, Xiong Lei, Hua Qiu, Tai-yuan Li

**Affiliations:** 1 Department of General Surgery, The First Affiliated Hospital of Nanchang University, No. 17 Yongwaizheng Street, Nanchang, 330006 Jiangxi China; 2 Medical College of Nanchang University, No. 461 Bayi avenue, Nanchang, 330006 Jiangxi China

**Keywords:** Advanced gastric cancer, Robotic-assisted gastrectomy, Laparoscopic-assisted gastrectomy, Short-term outcome

## Abstract

**Background:**

Robotic-assisted surgery, a developed technology, is becoming more and more accepted by surgeons. However, the comparison between robotic-assisted total gastrectomy (RATG) and conventional laparoscopy-assisted total gastrectomy (LATG) for advanced gastric cancer (AGC) is seldom reported, or usually the sample sizes reported are small. The current research was designed to compare the short-term outcomes of RATG and LATG with D2 lymphadenectomy for AGC in a mono-institution from China.

**Methods:**

A total of 205 patients from June 2015 to October 2018 were included in this study. Among them, 106 patients underwent LATG, and 99 patients underwent RATG. The patients’ clinicopathological characteristics, surgical performance and short-term outcomes were retrospectively analyzed.

**Results:**

The clinicopathological characteristics showed no difference between the LATG group and the RATG group. However, compared with the LATG group, the operation time was longer (*P* = 0.000), and the operative blood loss (*P* = 0.000) and the volume of abdominal drainage was less (*P* = 0.000) in the RATG group. Moreover, the RATG took less time to remove abdominal drainage tube than LATG (*P* = 0.000). The plasma levels of CRP at 72 h post-operation was lower (*P* = 0.000), and the number of retrieved lymph nodes was more (*P* = 0.000) in the RATG group. Nevertheless, the postoperative length of stay (*P* = 0.890), the time to first flatus (*P* = 0.448), the postoperative complication (*P* = 0.915) and the visual analogue pain score at 24 h post-operation (*P* = 0.457) were comparable between the two groups.

**Conclusions:**

RATG with D2 lymphadenectomy shows safety and feasibility for AGC and could be served as an alternative treatment for AGC in the future.

## Background

Gastric cancer is becoming a healthy problem worldwide with its increasing morbidity and mortality [1, 2]. Currently, it is generally known that the most effective therapeutics for the patients with AGC is partial or total gastrectomy with D2 lymphadenectomy. The appliance of laparoscopy-assisted gastrectomy (LAG) in gastric cancer was initially reported by Kitano et al. in 1994 [3]. Since then, laparoscopy has become more and more widely applied in the field of gastric cancer with its minimally invasive advantages [4–7]. Moreover, several researches have reported that LAG shows better short-term outcomes and equivalent long-term oncological outcomes for AGC when compared to the open gastrectomy [8–10]. However, LAG has several demerits, including the lack of spatial stereoscopic visualization, straight rigid instruments, amplified hand trembling, long learning curve and high demand for assistants and camera holder [11].

The surgical system of Da Vinci® (Intuitive Surgical, California, USA) was invented to overcome these technical difficulties. Since 2002 when Hashizume et al. [12] firstly described robotic-assisted gastrectomy for gastric cancer, multiple studies on comparing the short-term outcomes between LAG and RAG for gastric cancer were reported. However, most of the subjects were at a relatively early stage and the sample sizes were small [13, 14]. Thus, we conducted the present retrospective cohort study with a sample size including 205 patients for comparison of the short-term outcomes of RATG and LATG for AGC from a mono-institution in China.

## Methods

### Patients

From June 2015 to October 2018, 99 patients underwent RATG and 106 patients underwent LATG for AGC at the department of Gastrointestinal Surgery, The First Affiliated Hospital of Nanchang University. Patients had the freedom to choose their preferred approach after being informed the merits and demerits of both LATG and RATG which was introduced in 2014 by the surgeon. The patients with the following conditions were excluded from the study: 1) gastric stump cancer; 2) T-stage: pTis, pT1, or pT4b; 3) distant metastasis; 4) synchronous malignancy in other organs; 5) emergency operation; 6) serious cardiovascular or respiratory disorders, or hepaticorenal failure; 7) preoperative chemotherapy or radiation therapy. Furthermore, all of the patients were examined with electronic gastroscopy and thoracic and abdominal enhanced computed tomography (CT) scans to evaluate the preoperative tumor stage.

The clinicopathologic characteristics (gender, age, body mass index (BMI), tumor-node-metastasis (TNM) classification, tumor location and American Society of Anesthesiologists (ASA) Physical Status Classification,) and the short-term outcomes (operative blood loss, operation time, time-to-first flatus, postoperative morbidity, postoperative hospitalization time, pathological results, the visual analogue pain score at 24 h after surgical operation, the plasma levels of CRP at 72 h after operation and 24 h before surgery) of the patients were collected and compared between the two groups. Our research was approved by the institutional review of The First Affiliated Hospital of Nanchang University and the study compliance with the Helsinki Declaration. Written informed consents were obtained from all of the patients. The total gastrectomy was performed following the Japanese Gastric Cancer Treatment Guidelines [15] and the pathological stages of gastric cancer were classified in accordance with the cancer staging system of the 8th edition of American Joint Committee on Cancer (AJCC) [16].

### Surgical procedures

All of the RATG and LATG procedures were performed by a specific surgeon (TYL) with extensive experience in laparoscopic gastrectomy. All patients were conducted with endotracheal intubation and general anesthesia. During surgeries, the patients were placed in the supine and reverse Trendelenburg position with the legs elevated approximately 15°-20°and separated. Most of the operative steps in the RATG were the same as those in the LATG. Both operation procedures used a total of five trocars, adopts “W type” in RATG group and “U type” in LATG group. The camera port was inserted in the infraumbilical area by the closed method with a 12-mm trocar in RATG and a 10-mm trocar in LATG. Pneumoperitoneum was established with an intra-abdominal pressure of 12–15 mmHg by CO_2_ gas. Two 8-mm trocars for the first and the third robotic arm in RATG (one 12-mm trocar for operator and one 5-mm trocar for assistant in LATG) were placed in the left and right anterior axillary line just 2 cm below subcostal. One 8-mm trocar for second robotic arm and one 12-mm trocar for an assistant port in RATG (Two 5-mm trocars for operator and assistant in LATG) were placed through the left and right midclavicular line just 2 cm below (or above in LATG) umbilicus. The adjacent trocar distance is more than 8 cm to avoid interference between the manipulator. Standard D2 lymphadenectomy was performed in all procedures in accordance with the version 4 of Japanese Gastric Cancer Treatment Guidelines [15]. Afterward, the specimen was removed through a 6–8 cm mid-abdominal incision and Roux-en-Y esophagojejunostomy was performed with a 25 mm circular stapler. Seromuscular layer embedded was used to reinforce duodenal stump in all patients. Finally, two drainage tubes were placed near the duodenal stump and splenic recess respectively. The criteria to remove drainage tubes were the following: 1) the abdominal drainage volume less than 10 ml per day; 2) the drainage without odor; 3) patients without fever or peritonitis symptoms; 4) 4 days after operation.

### Statistical analysis

SPSS 22.0 software was used for the data analysis in the present study. Continuous variables were presented as mean ± SD when variables are normally distributed. If normal distribution failed to be assumed, the variables were presented as median and range. Continuous variables in normal distribution were compared between two groups using t-test otherwise Mann-Whitney U-test. For categorical variables presented as numbers and percentages, chi-squared test or Fisher’s exact test was used. *P* < 0.05 were considered as statistically significant.

## Results

### Clinicopathologic characteristics

Table 1 showed the clinicopathologic characteristics of the enrolled patients in the RATG and LATG groups. 113 male and 92 female patients with the average age of 58.9 years (range 43 to 75 years) were included in the present study. Age, gender, BMI, location of neoplasm, diameter of neoplasm, pathological Tumor stage, pathological Nodes stage or the TNM stage and ASA score showed no striking differences between the RATG group and LATG group (*P* > 0.05).

**Table 1:** Patient’s clinicopathological characteristics of RATG and LATG group for AGC

Characteristics	RATG (*n* = 99)	LATG (*n* = 106)	*P* value
Gender (n, %)			0.335
Male	58 (58.6)	55 (51.9)	
Female	41 (41.4)	51 (48.1)	
Age (mean ± SD, years)	58.7 ± 6.7	59.0 ± 7.3	0.743
Body mass index (median and range, kg/m^2^)	23.9 (17.3–28.6)	23.9 (19.9–28.3)	0.548
location of neoplasm (n, %)			0.350
Corpora ventriculi	66 (66.7)	64 (62.7)	
Fundus ventriculi	33 (33.3)	42 (37.3)	
T stage (n, %)			0.840
2	26 (26.3)	28 (26.4)	
3	40 (40.4)	39 (36.8)	
4	33 (33.3)	39 (36.8)	
N stage (n, %)			0.861
0	30 (30.3)	28 (26.4)	
1	30 (30.3)	29 (27.4)	
2	16 (16.2)	18 (17.0)	
3a	16 (16.2)	20 (18.9)	
3b	7 (7.1)	11 (10.4)	
pTNM (n, %)			0.376
1b	2 (2.0)	3 (2.8)	
2a	23 (23.2)	22 (20.8)	
2b	31 (31.3)	29 (27.4)	
3a	27 (27.3)	24 (22.6)	
3b	13 (13.1)	17 (16.0)	
3c	3 (3.0)	11 (10.4)	
ASA score (n, %)			0.900
1	52 (52.5)	53 (50.0)	
2	40 (40.4)	44 (41.5)	
3	7 (7.1)	9 (8.5)	
diameter of neoplasm (mm)	47.6 ± 12.7	47.0 ± 12.4	0.763

*RATG* robotic-assisted total gastrectomy, *LATG* laparoscopy-assisted total gastrectomy, *TNM* tumor node metastasis staging, *ASA* American Society of Anesthesiologists

### Intraoperative and postoperative outcomes

Table 2 showed the intraoperative and postoperative outcomes for the patients in the two groups. The RATG group was related with less operation blood loss (134.5 ± 12.9 vs. 152.8 ± 12.0 mL, *P* = 0.000) and longer operation time (203.9 ± 13.6 vs. 183.6 ± 12.1 min, *P* = 0.000) as compared with the LATG group. The volume of abdominal drainage was 338.0 (256.0–760.0) mL in the RATG group and 397.0 (253.0–705.0) mL in the LATG group (*P* = 0.000). The time to remove abdominal drainage tube was also shorter in the RATG group than in the LATG group (6.0 (5.0–27.0) vs. 7.0 (4.0–28.0) days, *P* = 0.000). Plasma level of CRP at 72 h after operation was lower in the RATG group than that in the LATG group (73.2 ± 8.0 vs. 78.7 ± 7.0 mg/L, *P* = 0.000). Furthermore, the number of harvested lymph nodes in RATG group was more than that in LATG group (25.8 ± 4.0 vs. 22.2 ± 3.8, *P* = 0.000). However, the postoperative length of stay, the time to first flatus and the visual analogue pain score at 24 h after operation were comparable between the two groups (*P*>0.05) (Table 2). The overall postoperative complication rates were similar in the two groups (*P* = 0.915), with 7.5 and 9.1% in the RATG and LATG groups respectively. There were eight complications in the RATG group and nine in the LATG group (Table 2).

**Table 2:** Operative outcomes of gastric cancer patients who underwent RATG or LATG

Operative outcomes	RATG (n = 99)	LATG (n = 106)	*P* value
Operative time (min)	203.9 ± 13.6	183.6 ± 12.1	0.000
Operative blood loss (mL)	134.5 ± 12.9	152.8 ± 12.0	0.000
Postoperative volume of abdominal drainage (mL)	338.0 (256.0–760.0)	397.0 (253.0–705.0)	0.000
Time to remove abdominal drainage tube (days)	6.0 (5.0–27.0)	7 (4.0–28.0)	0.000
Preoperative plasma levels of CRP (mg/L)	1.9 ± 0.8	2.0 ± 0.8	0.185
plasma levels of CRP at 72 h after operation (mg/L)	73.2 ± 8.0	78.7 ± 7.0	0.000
Numbers of retrieved lymph nodes (n)	25.8 ± 4.0	22.2 ± 3.8	0.000
Visual analogue pain score at 24 h after operation (scores)	6.0 (4.0–8.0)	5.0 (2.0–7.0)	0.457
Time to first flatus (hours)	55.5 ± 6.0	56.2 ± 7.5	0.448
Complications (n, %)	8 (7.5%)	9 (9.1%)	0.915
wound infection	2	3	1.000
pneumonia	2	1	0.952
deep vein thrombosis	1	0	0.483
intestinal obstruction	1	1	1.000
esophagojejunostomy anastomotic bleeding	1	0	0.483
duodenal stump leakage	1	1	1.000
esophagojejunostomy anastomotic leakage	0	1	1.000
chylous leakage	0	1	1.000
heart failure	0	1	1.000
Postoperative length of stay (days)	8.0 (6.0–30.0)	8.0 (5.0–29.0)	0.890

*CRP* C-reactive protein

## Discussion

LAG used for gastric cancer therapy was firstly reported by Japanese scholars in 1994 [3]. Since then, LAG has been increasingly used in many minimally invasive surgery centers around the world. However, the inherent properties of conventionally laparoscopic instruments limit its application in the treatment of gastric cancer, especially in lymph node dissection [17, 18]. The Da Vinci® robotic surgical system is a promising technique in overcoming some of limitations of LATG, has a qualitative leap forward and been applied more and more widely in the field of gastrointestinal surgery [19, 20]. However, the enrolled subjects rarely suffered from AGC in these studies. Besides, there is still scant information reported for the application of robotic surgery in AGC. Thus, we conducted this retrospective cohort research for the comparison of the short-term outcomes of RATG and LATG for AGC. Our results shows that RATG significantly decreased the operative blood loss, the level of plasma CRP at 72 h after operation, the postoperative volume of abdominal drainage and the time to remove abdominal drainage tubes. The study also reveals that RATG can dissect more lymph nodes. However, the RATG group is related with longer operative time than LATG group.

The operation time of RATG was significantly longer than that of LATG for AGC in this study, which was consistent with previous retrospective [11] and meta-analyses studies [21]. The longer operative time of RATG probably resulted from the robotic set-up time, the docking time and the time of camera motion interruption [22]. Some procedures in the RATG were performed by the assistant, such as applying hemoclips, inserting and removing surgical gauzes, which also significantly prolonged the operative time. Moreover, the operative blood loss was markedly reduced in the RATG group than that in LATG group. Several studies [23, 24] have reported similar results. This could be attributed to the fact that Da Vinci® robotic surgical system has eliminated tremor from operator, has provided three-dimensional stereoscopy and high degrees freedom EndoWrist, which could avoid the injuries to blood vessels such as the common hepatic artery, the celiac trunk and the splenic artery and allow precise dissection, especially in the inferior pylorus area and the superior area of pancreas.

The volume of abdominal drainage after the surgery may be a factor related to the quality of the operation. In the current research, we discovered that the volume of abdominal drainage after operation in the RATG group was less than that in the LATG group (*P* = 0.000). The drainage mainly resulted from exudates in the operation area. Robot has the advantages including highly flexible multi-joint forceps, high-definition and three-dimensional image and greatly improved ergonomics [25], which could keep mesentery such as mesogastrium and mesocolon transversum more perfect. Furthermore, Robotic could reduce the residual of adipose and lymphatic tissue around the stomach. Thus, Robot diminished the exudates and the volume of abdominal drainage. Therefore, the time to remove the abdominal drainage tube was shorter in the RATG group than that in the LATG group.

The level of plasma CRP at 72 h after operation may be another factor related to the quality of the operation. In the present study, the level of plasma CRP at 72 h postoperatively was dramatically lower in the RATG group than that in the LATG group. This may be explained by the fact that the surgical system of Da Vinci® is more stable and accurate in operation, which can reduce the irritation to normal tissues such as abdominal wall and bowl, maintain the integrity of mesentery, and also reduce the subsidiary-injury of the instruments.

Most patients with AGC have varied degrees of lymph node metastasis. D2 lymphadenectomy is one of the crucial steps in the surgery for AGC [15]. The number of lymph nodes obtained during operation is very important for judging the pathological stage and prognosis of patients [26]. As we all know, lymphadenectomy is difficult to be performed in the gastrectomy procedure, especially dissecting the lymph node around the field of the common hepatic artery, the splenic vessels and the ligamentum hepatoduodenale, which represent the mine field of D2 lymph node dissection. The current research revealed that RATG had the ability to retrieved more lymph nodes as compared with LATG (25.8 ± 4.0 vs. 22.2 ± 3.8, *P* = 0.000). Several studies have reported similar results with us [11, 27]. The main reasons are as follows: firstly, the Da Vinci® surgical system allowed the surgeon to reach deep-seated vessels and the delicate areas more easily. Secondly, robot traction and exposure are more stable than laparoscopy and may facilitate the difficult lymph node dissection in above areas. Thirdly, the robotic surgical field of vision is clearer than that of laparoscopy.

However, the postoperative length of stay, the visual analogue pain score at 24 h after the operation and the time to first flatus did not differ between the RATG group and LATG group (*P* > 0.05). The comparable postoperative length of stay between the two groups may be attributed to the postoperative complications and the patient’s discharge willingness in our center. Postoperative pain was mainly caused by the incision. In our center, removal of whole stomach specimen and anastomosis were conducted by a 6- to 8-cm median superior abdominal incision both in the RATG and LATG group. This was the possible reason why visual analogue pain score at 24 h after the operation did not differ between two groups. The time to the first flatus had similar results between the two groups, which might be because gastrectomy was mainly on supracolic compartment, rarely irritated and stimulated bowel. The postoperative complications were important when assessing the quality and safety of operations. In the current study, complication rates was found no obvious difference between the RATG and LATG groups (7.5% vs 9.1%, *P* = 0.915).

The differences in our study were comparable as all procedures were performed by one surgeon (TYL) and may become even more obvious in the future as the surgeon will be more experienced in RATG. At the same time, we admit that this study is neither a randomized controlled trial nor a prospective analysis study, but a retrospective study. Therefore, there exists some bias on sample selection. Another limitation of the current study is that we didn’t compare the costs of the two surgeries. Further research especially the high-quality randomized controlled studies are needed.

## Conclusions

In conclusion, the current research illustrated that RATG with D2 lymphadenectomy for AGC was a feasible and safe surgical procedure. Compared with LATG, RATG showed lower plasma levels of CRP 72 h after operation, less operative blood loss, less volume of abdominal drainage after operation and shorter time to remove abdominal drainage tube. More high-quality randomized controlled studies remain to be required for the applications.

## Data Availability

Access to the database can be obtained from the corresponding author on reasonable request.

## Abbreviations

AGC: Advanced gastric cancer
ASA: American Society of Anesthesiologists
BMI: Body mass index
CRP: C-Reactive protein
LAG: Laparoscopy-assisted gastrectomy
LATG: Laparoscopy-assisted total gastrectomy
RATG: Robotic-assisted total gastrectomy
SD: Standard Deviation
TNM: Tumor-node-metastasis
